# Characterization of three cell lines derived from fine needle biopsy of choroidal melanoma with metastatic outcome

**Published:** 2011-02-25

**Authors:** Barry L. Burgess, Nagesh P. Rao, Ascia Eskin, Stanley F. Nelson, Tara A. McCannel

**Affiliations:** 1 Department of Ophthalmology, University of California, Los Angeles, The Jules Stein Eye Institute, Los Angeles, CA; 2 Department of Pathology and Laboratory Medicine, University of California, Los Angeles, Los Angeles, CA; 3 Department of Human Genetics, University of California, Los Angeles, Los Angeles, CA

## Abstract

**Purpose:**

To report three low-passage cell lines from primary choroidal melanoma with metastatic outcome, which were stable for cytogenetic patterns and expression profiles of the primary melanoma.

**Methods:**

In patients with choroidal melanoma, transscleral fine needle aspiration biopsy (FNAB) was performed immediately before plaque placement for ^125^iodine brachytherapy or immediately after enucleation. Cells were examined for cytopathology, evaluated by fluorescence in-situ hybridization (FISH) for the centromere of chromosome 3, analyzed by 250K whole genome Mapping Array and U133 plus 2.0 Expression Array, and placed in cell culture. At passage 3, the cell lines were analyzed by Mapping Array and Expression Array.

**Results:**

Three cell lines were propagated from primary choroidal melanomas in three patients who subsequently developed metastasis. Two cell lines were stable for the entire chromosomal aberration pattern of the respective primary tumor. In the third, necrotic material from the biopsy prevented further analysis, yet resulted in a stable cell line. Each cell line had chromosome 3 loss, 6q loss, 8p loss, multiple 8q gain, and 16q loss. Additionally, two cell lines had chromosome 6p gain. Two cell lines had RNA expression profiles similar to the respective primary tumors; the third cell line had a similar RNA expression profile relative to the other two cell lines.

**Conclusions:**

FNAB of primary choroidal melanomas resulted in highly characterized, low-passage cell lines, which were stable for the cytogenetic patterns and expression profiles found in the primary tumor. These cell lines represent novel tools for the study of metastatic choroidal melanoma biology.

## Introduction

Choroidal melanoma (melanoma arising primarily from the ciliary body or choroid) is the most common primary intraocular malignancy in adults. Despite treatment with brachytherapy, external beam radiation or enucleation, long-term follow-up is associated with melanoma-related death in approximately 50% of patients [1].

Most strongly correlated with increased risk of metastasis are cytogenetic aberrations and gene expression abnormalities in melanoma cells [2-5]. Loss of one copy of chromosome 3 (monosomy 3), loss of one copy of chromosome 8p and class II gene expression profile are most strongly associated with a high-risk of metastasis [4,6-10].

Melanoma cytogenetic abnormalities provide information regarding the fundamental molecular biology of choroidal melanoma and may uncover potential genes for targeted therapy [11,12]. Recognizing the potential value of studying melanoma cytogenetic aberrations and associated gene expression, this report presents development and characterization of low-passage choroidal melanoma cell lines that were stable for the cytogenetic patterns and gene expression profiles found in primary choroidal melanomas that resulted in metastatic disease.

## Methods

All studies were performed in accordance with the United States Health Insurance Portability and Accountability Act (HIPAA) of 1996. All participants gave informed consent and all studies were approved by the Office of the Human Research Protection Program (Institutional Review Board) of the University of California, Los Angeles, Los Angeles, CA.

### Tissue collection

In patients with primary choroidal melanoma and no clinical evidence of metastasis, transscleral fine needle aspiration biopsy (FNAB) was performed immediately before plaque placement for ^125^iodine brachytherapy or immediately after enucleation. As described elsewhere, cells obtained from the biopsy were examined for cytopathology, evaluated by fluorescence in-situ hybridization (FISH) for the centromere of chromosome 3, analyzed by 250K Mapping Array (Affymetrix, Santa Clara, CA) for chromosomal copy number variation, U133 plus 2.0 Arrays (Affymetrix) for gene expression, and placed in cell culture [11-13].

### Cell culture

Triturated biopsy cells were cultured in a growth medium of DMEM, 10% human AB serum, 5 µg/ml bovine insulin and glutamine-pen-strep at 37 °C, 7% CO_2_. Primary cultures were seeded in 12.5 cm^2^ vented flasks and grown to confluency (about 12 weeks) replacing half of the medium every 3 days.

These spontaneous cultures were expanded into 75 cm^2^ flasks and evaluated at passage 3. Briefly, trypsinized cells were stabilized in RNAprotect Cell Reagent (Qiagen, Valencia, CA) and analyzed by 250K Mapping Array and U133 plus 2.0 Expression Array (Affymetrix) as described hereafter. Beginning at passage 3, portions of the cell lines were cryogenically preserved for use in future studies and passaged in cell culture to document the characteristics of continued proliferation.

### Nucleic acid analyses

Genomic DNA and total RNA were sequentially isolated from trypsinized cell cultures using an AllPrep DNA/RNA Mini Kit (Qiagen). Isolated DNA was quantified using a NanoDrop ND-1000 Spectrophotometer (NanoDrop, Wilmington, DE). No DNA sample was subjected to whole genome amplification techniques. DNA copy number was assessed using GeneChip Human 250K NSPI Mapping Arrays (Affymetrix). Probe preparation, hybridization, and reading were performed by the UCLA DNA Microarray Core according to the standard 96-well protocol published by Affymetrix. Copy number variation was computed using Genome Console software from Affymetrix [11].

RNA was quantified on a NanoDrop Spectrophotometer and analyzed on a 2100 Bioanalyzer (Agilent, Santa Clara, CA) for integrity. RNA had an A260/280 ratio of >1.90 and RNA integrity number (RIN) of 8.5 or higher as determined by the 2100 Bioanalyzer. Prepared RNA was hybridized to GeneChip Human Genome U133 Plus 2.0 Arrays (Affymetrix) at the UCLA DNA Microarray Core Facility using the standard Affymetrix protocol.

### GNAQ mutation

Additionally, the nucleotide sequence of exon 5 of the guanine nucleotide binding protein (G protein) q polypeptide (*GNAQ*) was determined for both cell line genomic DNA and available germ line DNA from blood samples via PCR and sequencing on an ABI 3130 Genetic Analyzer using BigDye 2.0 chemistry (Life Technologies, Carlsbad, CA), and intronic sequence primers 5′-TTC CCT AAG TTT GTA AGT AGT GC-3′ and 5′-AGA AGT AAG TTC ACT CCA TTC C-3′ encompassing exon 5 of *GNAQ*.

## Results

Cell cultures from choroidal melanomas with chromosome 3 loss (monosomy 3) showed variable propagation (Table 1). Among choroidal melanomas with chromosome 6p gain in the absence of chromosome 3 loss, all cell cultures failed to propagate under the growth conditions employed. Each of the three cell lines were derived from two pooled fine needle aspirates containing approximately 110, 500, or 350 viable cells for MEL20–06–039, MEL20–06–045, or MEL20–07–070, respectively, as determined by counts of adherent cells obtained 24 h after initial seeding in a 12.5 cm^2^ flask. In each case epithelioid morphology was initially observed. Macrophages and erythrocytes were present initially, but rapidly degraded. No fibroblasts were observed. At confluency, each culture was expanded into a 75 cm^2^ flask. All subsequent passages were split 1:3 in 75 cm^2^ flasks with 1×10^7^ cells removed and aliquoted for cryopreservation.

**Table 1:** Choroidal melanoma cell cultures.

**59 Melanomas with Chromosome 3 Loss**	**Number**
Successful propagation and characterization of cell lines	3 (5%)
Successful early propagation but not yet characterized	6 (10%)
Failure to propagate beyond first passage	40 (68%)
Failure to propagate	10 (17%)
**42 Melanomas with Chromosome 6p Gain/Disomy 3**	Number
Failure to propagate	42 (100%)

**Figure 1:**
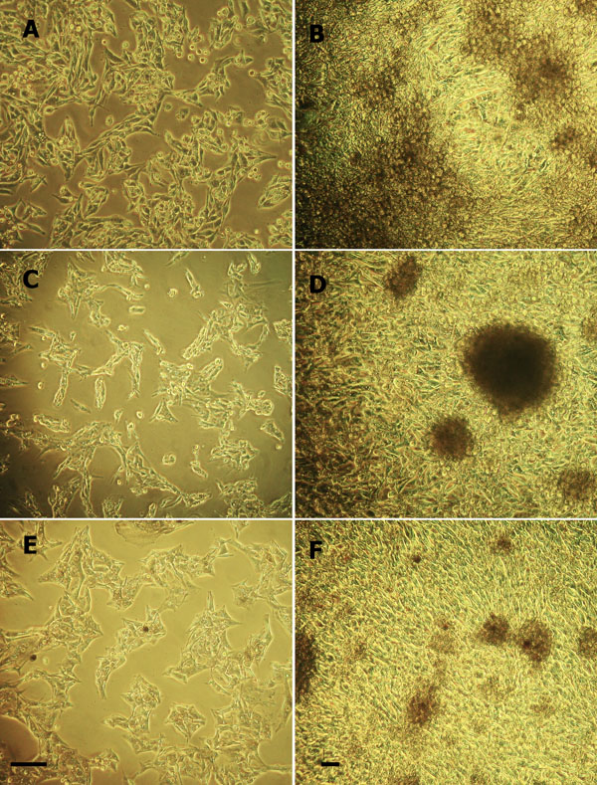
Phase contrast photomicrographs of cell lines in subconfluent and overgrowth conditions. At passage 3, cell lines MEL20–06–039 (**A** and **B**), MEL20–06–045 (**C** and **D**) and MEL20–07–070 (**E** and **F**) show early stage propagation (**A**, **C**, and **E**) and late stage confluent propagation with heaped up cells and no evidence of contact inhibition (**B**, **D**, and **F**). Melanin production is evident in two cell lines (MEL20–06–045 and MEL20–07–070). Original magnification (**A**, **C**, **E**) 200×, (**B**, **D**, **F**) 100×. Scale bar is 30 µm.

The three characterized cell lines with monosomy 3 were from patients with choroidal melanoma who developed clinical evidence of melanoma metastasis <1.5 years after primary melanoma diagnosis and treatment; all three patients subsequently died from liver failure due to metastatic disease. All three cell lines demonstrated stable propagation through at least passage 6 over a period of 2.5 years. Two of the three cell lines persisted in melanin production beyond passage 6 and none of the cultures exhibited contact inhibition in over-growth conditions (Figure 1).

**Figure 2:**
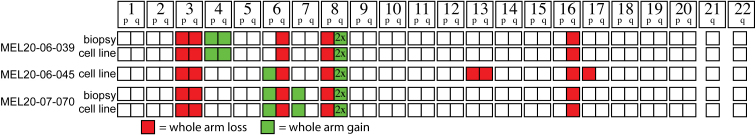
Comparison of Mapping Array results for chromosomal copy number for primary tumor biopsies and their cell lines, where available. Copy number variation analysis, as determined by Affymetrix Genotyping Console, showed identical patterns of aberration between primary tumor biopsies and corresponding cell lines. Gains (green boxes) and losses (red boxes) were largely whole arm in extent. 2± designations signify a total of 4 or more copies of chromosome 8q were detected. All samples had monosomy 3, 6q loss, 8p loss, multiple gains in 8q, and 16q loss. MEL20–06–045 and MEL20–07–070 also had 6p gain.

**Figure 3:**
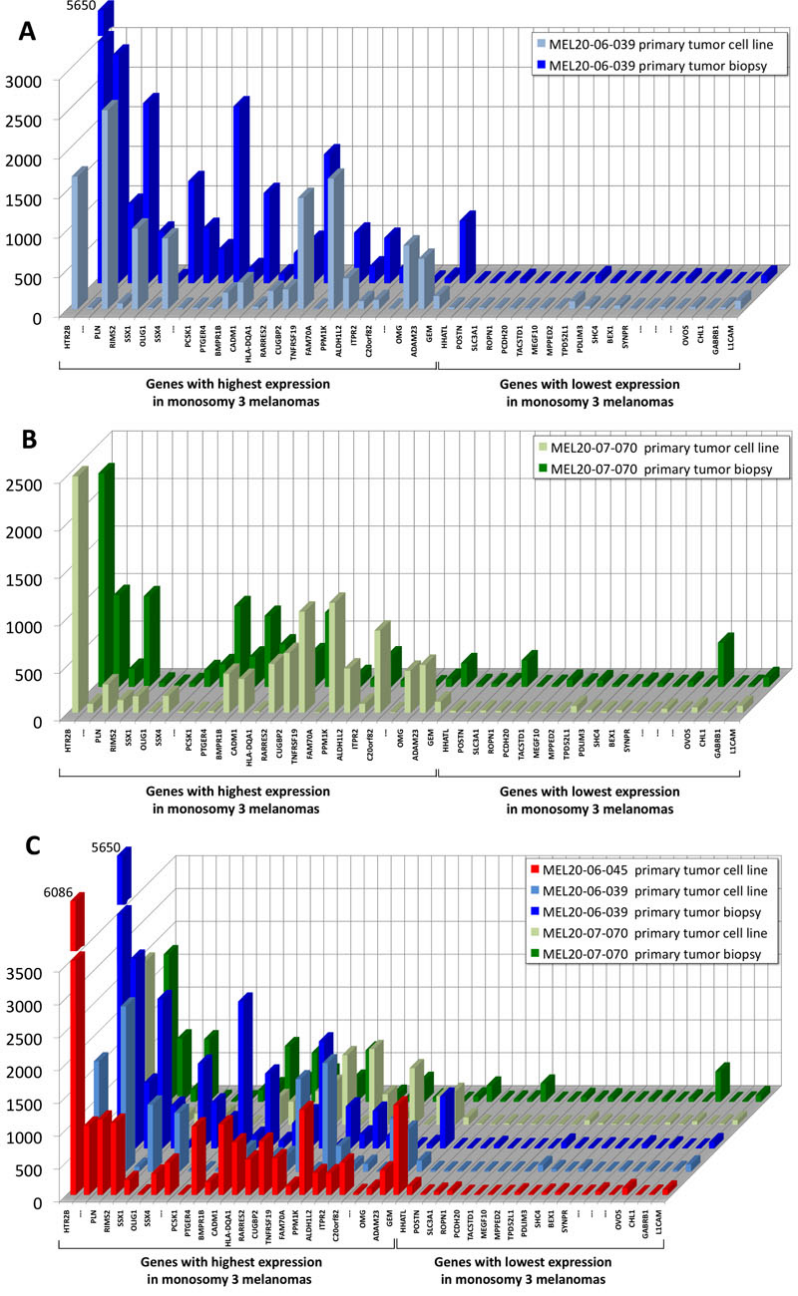
Comparison of expressional characteristics between primary melanomas and their corresponding cell lines. Normalized expressional values from Affymetrix GeneChip HG U133 plus 2.0 arrays for 25 genes significantly upregulated in monosomy 3 tumors and 20 genes significantly down-regulated or silenced in monosomy 3 melanomas compared to melanomas with 6p gain in the absence of chromosome 3 loss were selected to compare expressional variation between primary tumor biopsies and the corresponding cell lines. **A**: MEL20–06–039; **B**: MEL20–07–070; **C**: MEL20–06–045 cell line is shown in combination with MEL20–06–039 and MEL20–07–070 to provide a comparison of this cell line’s expressional profile with those for which primary tumor expressional characteristics are known. Probeset identifiers and genomic locations of the listed genes are detailed in Table 2.

Cytogenetically, two of the three cell lines (MEL20–06–039 and MEL20–07–070) demonstrated the chromosomal aberration pattern present in the respective primary melanoma, including monosomy 3, 6q loss, 8p loss, multiple 8q gain and 16q loss (Figure 2). The third cell line (MEL20–06–045), derived from a biopsy that showed necrotic material, had a chromosomal aberration pattern that was nearly identical with the other two cell lines. Additionally, the MEL20–06–045 cell line and both the MEL 20–07–070 cell line and primary tumor exhibited chromosome 6p gain. Moreover, two of the cell lines (MEL20–06–039 and MEL20–07–070) had RNA expression array characteristics similar to the respective primary tumor for the 25 most overexpressed and the 20 most under-expressed genes from a comparative gene list composed of genes most overexpressed and under-expressed in an integrated analysis of choroidal melanoma with chromosome 3 loss versus 6p gain in the absence of chromosome 3 loss (Figure 3A,B) [11]. The third cell line (MEL20–06–045) had a similar profile of gene expression when compared to the two primary melanomas and the other two cell lines (Figure 3C).

**Table 2:** Genes upregulated, down-regulated, or silenced in monosomy 3 tumors.

**Genes significantly upregulated in monosomy 3 tumors**		
**Probe set ID**	**Gene symbol**	**Location**
206638_at	*HTR2B*	chr2q37.1
229823_at	—	chr8q22.3
204939_s_at	*PLN*	chr6q22.31
206137_at	*RIMS2*	chr8q22.3
206626_x_at	*SSX1*	chrXp11.22
228170_at	*OLIG1*	chr21q22.11
211425_x_at	*SSX4*	chrXp11.23
230650_at	—	chr8q13.3
205825_at	*PCSK1*	chr5q15
204897_at	*PTGER4*	chr5p13.1
229975_at	*BMPR1B*	chr4q22.3
209031_at	*CADM1*	chr11q23.2
212671_s_at	*HLA-DQA1*	chr6p21.32
209496_at	*RARRES2*	chr7q36.1
202158_s_at	*CUGBP2*	chr10p14
227812_at	*TNFRSF19*	chr13q12.12
219895_at	*FAM70A*	chrXq24
235061_at	*PPM1K*	chr4q22.1
231202_at	*ALDH1L2*	chr12q23.3
202660_at	*ITPR2*	chr12p11.23
235182_at	*C20orf82*	chr20p12.1
243339_at	—	chr5q33.1
238720_at	*OMG*	chr17q11.2
244463_at	*ADAM23*	chr2q33.3
204472_at	*GEM*	chr8q22.1
**Genes significantly down-regulated or silenced in monosomy 3 tumors**		
**Probe set ID**	**Gene symbol**	**Location**
223572_at	*HHATL*	chr3p22.1
210809_s_at	*POSTN*	chr13q13.3
205799_s_at	*SLC3A1*	chr2p21
231535_x_at	*ROPN1*	chr3q21.1
232054_at	*PCDH20*	chr13q21.31
201839_s_at	*TACSTD1*	chr2p21
232523_at	*MEGF10*	chr5q23.2
205413_at	*MPPED2*	chr11p14.1
203786_s_at	*TPD52L1*	chr6q22.31
238592_at	*PDLIM3*	chr4q35.1
235238_at	*SHC4*	chr15q21.1
218332_at	*BEX1*	chrXq22.1
230303_at	*SYNPR*	chr3p14.2
236936_at	—	chr8p11.23
236300_at	—	chr12p12.2
242206_at	—	chr7q11.21
228245_s_at	*LOC100132881*	chr12p13.31
234583_at	*CHL1*	chr3p26.3
207010_at	*GABRB1*	chr4p12
204584_at	*L1CAM*	chrXq28

**Figure 4:**
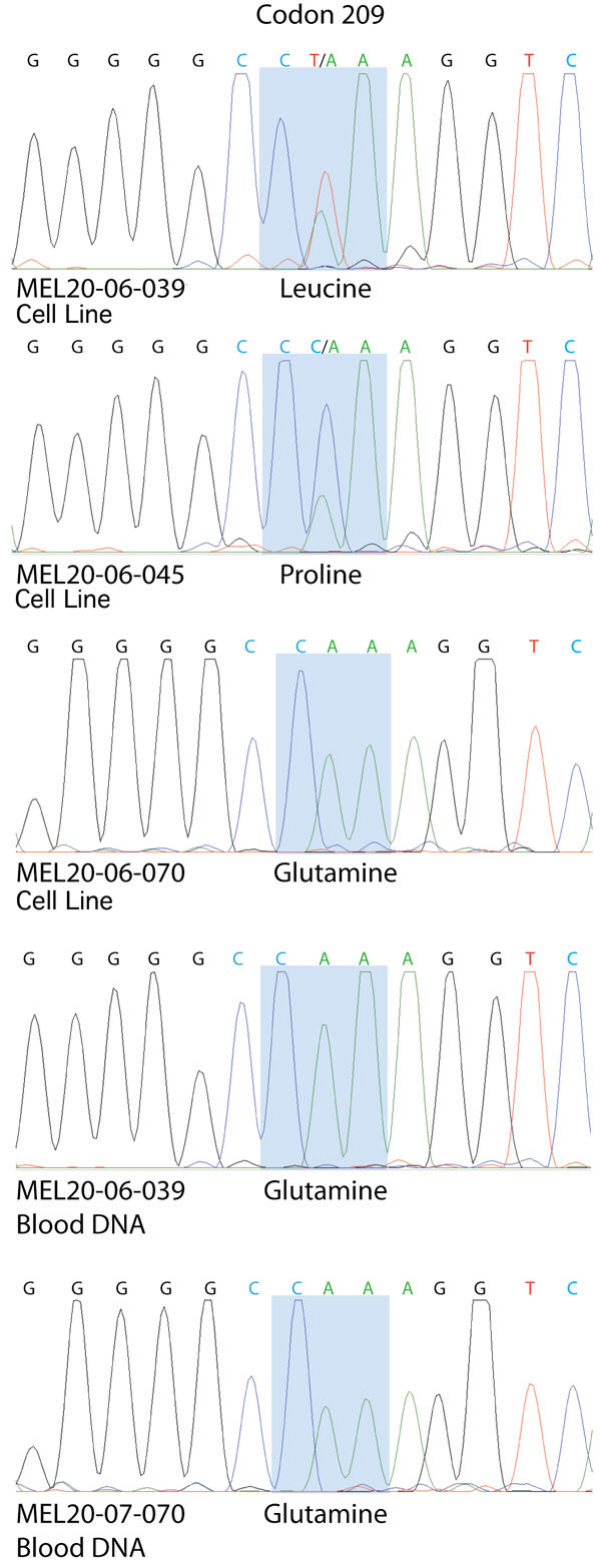
Sequence analysis of codon 209 of *GNAQ*. Heterozygous somatic mutations of Q209L and Q209P were identified in MEL20–06–039 and MEL20–06–045 cell lines, respectively. The MEL20–07–070 cell line was wild-type as were blood samples from the patients MEL20–06–039 and MEL20–07–070. No blood sample was available from patient MEL20–06–045.

Sequence analysis of exon 5 of *GNAQ* demonstrated heterozygous mutations in codon 209 in two of the three cultures (resulting in either Q209L or Q209P) and wild-type sequence in the third culture (Figure 4). Codon 209 sequence analysis of blood samples from the patients with Q209L and wild-type sequence revealed only wild-type sequence in both patients. No blood sample was available from the patient with Q209P gene mutation.

## Discussion

This report presents for the first time, to our knowledge, cell lines characterized by microarray and grown from choroidal melanoma in patients who developed clinical metastases. Cell line characterization at passage 3 with 250K Mapping Arrays showed that each of the three cell lines and the two respective primary melanomas had common cytogenetic characteristics for monosomy 3, 6q loss, 8p loss, multiple 8q gain, and 16q loss. Two of the cell lines, MEL20–06–045 and MEL20–07–070 also demonstrated gains in chromosome 6p. Analysis of gene expression with U133 Plus 2.0 Arrays showed that two of the cell lines expressed gene characteristics similar to the respective primary melanoma for the 25 most overexpressed genes and the 20 most under-expressed genes in a comparative gene list composed of genes most overexpressed and under-expressed in an integrated analysis of choroidal melanoma with chromosome 3 loss versus 6p gain in the absence of chromosome 3 loss [11]; the third cell line had a profile of gene expression similar to the two primary melanomas and the other two cell lines. Potentially relevant functional classes of biologic activity include G-protein coupled signaling, calcium response pathways, cell adhesion marker expression, retinoic acid response pathways and regulation of palmitoylation [11].

Furthermore, sequence analysis from exon 5 of *GNAQ* demonstrated heterozygous mutation of codon 209 in two of the three cell lines and the wild-type sequence in the third. *GNAQ* codon 209 mutations in choroidal melanoma have been shown to be important in primary melanoma tumor development [14,15].

Choroidal melanoma cell lines derived from melanomas that progressed to clinical metastases and patient death with cytogenetic characteristics and gene expression profiles closely corresponding to the respective primary melanoma are important for at least two reasons. First, there is no animal model of spontaneous uveal melanoma that accurately replicates the course of the human disease [16]. Transgenic animal models of human disease do not reproduce the histiogenesis of human melanoma and animal melanoma differs substantially from human uveal melanoma [17,18].

Second, cancer evolves through a process of step-wise accumulation of genetic alterations that result in uncontrolled cell proliferation and a lack of response to normal apoptotic stimuli. Genetic alterations include deletions, duplications/amplifications, translocations and point mutations that cause loss-of-function or gain-in-function and lead to altered protein expression. Availability of cell lines that closely correspond to the cells of metastasis-causing choroidal melanomas may serve as important in vitro models for the study of melanoma biology [19].

Characterization of the choroidal melanoma cell lines in this report extends the initial attempt at uveal melanoma cell culture by Kirby [20] in 1929, the report of six continuous cell lines from choroidal and ciliary body melanomas by Albert et al. [21]. in 1984 and subsequent reports of cell lines derived from uveal melanomas [22-28]. Cell lines from primary ciliary body and choroidal melanomas have been characterized by cellular morphology, relative growth rate, vasculogenic cord formation, immunohistochemistry, major histocompatability complex (MHC), and cytogenetic analysis of cell lines with karyotype study and determination of monosomy 3 status by microsatellite analysis. Cell lines derived from ciliary body and choroidal melanoma metastases have been studied by Luyton et al. [25,27] using similar methods including karyotype analysis. Adding an additional dimension, this report presents analysis of primary choroidal melanomas and their cell lines with high resolution microarrays to compare cytogenetic characteristics and gene expression profiles.

Recognition that cancer is fundamentally a genetic disease emphasizes the importance of precisely characterizing the cytogenetic and gene expression of primary choroidal melanomas and the corresponding cell lines. Choroidal melanoma cell lines with genetic alterations that reflect their primary tumor provide malleable in vitro models. These models enable mechanistic dissection of melanoma biology, identification of “driver” genes and insight regarding therapeutic targets [29].

Strengths of the three cell lines described in this report for in vitro study of choroidal melanoma biology and testing of therapeutic targets relate to (1) origin from primary choroidal melanomas that developed clinical evidence of metastasis <1.5 years after treatment of the primary tumor, (2) demonstration of cellular propagation through at least 6 passages, (3) documentation of equivalent cytogenetic aberrations and similar gene expression patterns in the primary melanomas and in the cell lines at passage 3, and (4) preservation of cell lines by freezing at passage 3 to provide highly characterized cell lines for future studies.

Limitations of the three cell lines as surrogates for study of melanoma biology, metastasis and therapy relate to (1) the limited but potentially significant differences in chromosomal aberration and gene expression between primary melanomas and corresponding cell lines, (2) the likelihood that, with long-term propagation and multiple passages, cell lines will undergo culture-related evolution in both chromosomal structure and gene expression, and (3) recognition that cancer biology is influenced by the in vivo micro-environment that is not replicated with in vitro models. However, the fact that the cell lines differ slightly between themselves may not be as important as having a diverse genetic background under which therapeutic drugs may be tested to determine biologic pathways in tumor development.

In summary, FNAB of primary choroidal melanomas that developed clinical metastases resulted in the development of three low-passage, highly characterized cell lines that exhibited the chromosomal aberrations and gene expression profiles present in the primary choroidal melanomas. These cell lines, with portions frozen beginning at passage 3 and portions continuing to propagate, provide in vitro models for study of choroidal melanoma biology, metastasis and therapy.

## References

[1] Kujala E Makitie T Kivela T Very long-term prognosis of patients with malignant uveal melanoma. Invest Ophthalmol Vis Sci 2003 44 4651 9 14578381 10.1167/iovs.03-0538

[2] Prescher G Bornfeld N Hirche H Horsthemke B Jöckel KH Becher R Prognostic implications of monosomy 3 in uveal melanoma. Lancet 1996 347 1222 5 8622452 10.1016/s0140-6736(96)90736-9

[3] White VA Chambers JD Courtright PD Chang WY Horsman DE Correlation of cytogenetic abnormalities with the outcome of patients with uveal melanoma. Cancer 1998 83 354 9 9669819

[4] Tschentscher F Hüsing J Hölter T Kruse E Dresen IG Jöckel KH Anastassiou G Schilling H Bornfeld N Horsthemke B Lohmann DR Zeschnigk M Tumor classification based on gene expression profiling shows that uveal melanomas with and without monosomy 3 represent two distinct entities. Cancer Res 2003 63 2578 84 12750282

[5] Sisley K Tattersall N Dyson M Smith K Mudhar HS Rennie IG Multiplex fluorescence in situ hybridization identifies novel rearrangements of chromosome 6, 15, and 18 in primary uveal melanoma. Exp Eye Res 2006 83 554 9 16684523 10.1016/j.exer.2006.02.007

[6] Worley LA Onken MD Person E Robirds D Branson J Char DH Perry A Harbour JW Transcriptomic versus chromosomal prognostic markers and clinical outcome in uveal melanoma. Clin Cancer Res 2007 13 1466 71 17332290 10.1158/1078-0432.CCR-06-2401

[7] Petrausch U Martus P Tönnies H Bechrakis NE Lenze D Wansel S Hummel M Bornfeld N Thiel E Foerster MH Keilholz U Significance of gene expression analysis in uveal melanoma in comparison to standard risk factors for risk assessment of subsequent metastases. Eye 2008 22 997 1007 17384575 10.1038/sj.eye.6702779

[8] van Gils W Lodder EM Mensink HW Kiliç E Naus NC Brüggenwirth HT van Ijcken W Paridaens D Luyten GP de Klein A Gene expression profiling in uveal melanoma: Two regions on 3p related to prognosis. Invest Ophthalmol Vis Sci 2008 49 4254 62 18552379 10.1167/iovs.08-2033

[9] Mensink HW Kiliç E Vaarwater J Douben H Paridaens D de Klein A Molecular cytogenetic analysis of archival uveal melanoma with known clinical outcome. Cancer Genet Cytogenet 2008 181 108 11 18295662 10.1016/j.cancergencyto.2007.12.001

[10] Damato B Dopierala J Klaasen A van Dijk M Sibbring J Coupland SE Multiplex ligation-dependent probe amplification of uveal melanoma: correlation with metastatic death. Invest Ophthalmol Vis Sci 2009 50 3048 55 19182252 10.1167/iovs.08-3165

[11] McCannel TA Burgess BL Rao NP Nelson SF Straatsma BR Identification of candidate tumor oncogenes by integrative molecular analysis of choroidal melanoma fine-needle aspiration biopsy specimens. Arch Ophthalmol 2010 128 1170 7 20837802 10.1001/archophthalmol.2010.180

[12] McCannel TA Burgess BL Nelson SF Eskin A Straatsma BR Genomic identification of significant targets in ciliochoroidal melanoma. Invest Ophthalmol Vis Sci 2010 20688739 10.1167/iovs.10-5864

[13] Young TA Burgess BL Rao NP Gorin MB Straatsma BR High density genome array is superior to fluorescence in-situ hybridization analysis of monosomy 3 in choroidal melanoma fine needle aspiration biopsy. Mol Vis 2007 13 2328 33 18199974

[14] Van Raamsdonk CD Bezrookove V Green G Bauer J Gaugler L O'Brien JM Simpson EM Barsh GS Bastian BC Frequent somatic mutations of GNAQ in uveal melanoma and blue naevi. Nature 2009 457 599 602 19078957 10.1038/nature07586 PMC2696133

[15] Onken MD Worley LA Long MD Duan S Council ML Bowcock AM Harbour JW Oncogenic mutations in GNAQ occur early in uveal melanoma. Invest Ophthalmol Vis Sci 2008 49 5230 4 18719078 10.1167/iovs.08-2145 PMC2634606

[16] Folberg R Kadkol SS Frenkel S Valyi-Nagy K Jager MJ Pe'er J Maniotis AJ Authenticating cell lines in ophthalmic research laboratories. Invest Ophthalmol Vis Sci 2008 49 4697 701 18689700 10.1167/iovs.08-2324 PMC2576485

[17] Tolleson WH Doss JC Latendresse J Warbritton AR Melchior WB Jr Chin L Dubielzig RR Albert DM Spontaneous uveal amelanotic melanoma in transgeneric Tyr-RAS+ Ink4a/Arf−/− mice. Arch Ophthalmol 2005 123 1088 94 16087843 10.1001/archopht.123.8.1088

[18] Wilcock BP Peiffer RL Jr Morphology and behavior of primary ocular melanomas in 91 dogs. Vet Pathol 1986 23 418 24 3750735 10.1177/030098588602300411

[19] Stuart D Sellers WR Linking somatic genetic alterations in cancer to therapeutics. Curr Opin Cell Biol 2009 21 304 10 19328671 10.1016/j.ceb.2009.02.001

[20] Kirby DB Tissue culture in ophthalmic research. Trans Am Ophthalmol Soc 1929 27 33 PMC1316743 16692839

[21] Albert DM Ruzzo MA McLaughlin MA Robinson NL Craft JL Epstein J Establishment of cell lines of uveal melanoma. Invest Ophthalmol Vis Sci 1984 25 1284 99 6386741

[22] Kan-Mitchell J Mitchell MS Rao N Liggett PE Characterization of uveal melanoma cell lines that grow as xenografts in rabbit eyes. Invest Ophthalmol Vis Sci 1989 30 829 34 2722439

[23] Aubert C Rouge F Reillaudou M Metge P Establishment and characterization of human ocular melanoma cell lines. Int J Cancer 1993 54 784 92 8325707 10.1002/ijc.2910540513

[24] De Waard-Siebinga I Blom DJ Griffioen M Schrier PI Hoogendoorn E Beverstock G Danen EH Jager MJ Establishment and characterization of a uveal-melanoma cell line. Int J Cancer 1995 62 155 61 7622289 10.1002/ijc.2910620208

[25] Luyten GP Naus NC Mooy CM Hagemeijer A Kan-Mitchell J Van Drunen E Vuzevski V De Jong PT Luider TM Establishment and characterization of primary and metastatic uveal melanoma cell lines. Int J Cancer 1996 66 380 7 8621261 10.1002/(SICI)1097-0215(19960503)66:3<380::AID-IJC19>3.0.CO;2-F

[26] Nareyeck G Zeschnigk M Prescher G Lohmann DR Anastassiou G Establishment and characterization of two uveal melanoma cell lines derived from tumors with loss of one chromosome 3. Exp Eye Res 2006 83 858 64 16750193 10.1016/j.exer.2006.04.004

[27] Zuidervaart W Hensbergen PJ Wong MC Deelder AM Tensen CP Jager MJ Gruis NA Proteomic analysis of uveal melanoma reveals novel potential markers involved in tumor progression. Invest Ophthalmol Vis Sci 2006 47 786 93 16505008 10.1167/iovs.05-0314

[28] Nareyeck G Zeschnigk M Bornfeld N Anastassiou G Novel cell lines derived by long-term culture of primary uveal melanomas. Ophthalmologica 2009 223 196 201 19212147 10.1159/000201566

[29] Lin WM Baker AC Beroukhim R Winckler W Feng W Marmion JM Laine E Greulich H Tseng H Gates C Hodi FS Dranoff G Sellers WR Thomas RK Meyerson M Golub TR Dummer R Herlyn M Getz G Garraway LA Modeling genomic diversity and tumor dependency in malignant melanoma. Cancer Res 2008 68 664 73 18245465 10.1158/0008-5472.CAN-07-2615 PMC10493008

